# Complement Modulation Mitigates Inflammation-Mediated Preterm Birth and Fetal Neural Inflammation

**DOI:** 10.3390/cells14141045

**Published:** 2025-07-08

**Authors:** Eliza R. McElwee, Devin Hatchell, Mohammed Alshareef, Khalil Mallah, Harriet Hall, Hannah Robinson, Ramin Eskandari, Eugene Chang, Scott Sullivan, Stephen Tomlinson

**Affiliations:** 1 Department of Obstetrics and Gynecology, Division of Maternal-Fetal Medicine, Medical University of South Carolina, Charleston, SC 29425, USA; rodrie@musc.edu (E.R.M.);; 2 Department of Pharmacology and Immunology, Medical University of South Carolina, Charleston, SC 29425, USA; 3 Department of Neurological Surgery, Division of Pediatrics, Medical University of South Carolina, Charleston, SC 29425, USA; 4 College of Medicine, Medical University of South Carolina, Charleston, SC 29425, USA; qxg7mj@uvahealth.org (H.H.); hannah.janelle.robinson@emory.edu (H.R.); 5 Ralph Johnson VA Medical Center, Charleston, SC 29425, USA

**Keywords:** complement system, complement modulation, preterm birth, fetal inflammation, maternal-fetal interface

## Abstract

Preterm birth and the neonatal pathological sequelae that follow spontaneous preterm labor are closely associated with maternal and fetal inflammatory activation. Previous studies have indicated a role for the complement system in this inflammatory response. Utilizing an LPS inflammation-induced model of preterm birth, we investigated various delivery outcomes and their correlation with complement activation products within cervical, uterine, and fetal brain tissue after administration of LPS. We provide further evidence that complement-mediated inflammation within cervical and uterine tissue contributes to aberrant cellular changes and an increase in preterm delivery. We additionally show that a targeted complement inhibitor that specifically targets to sites of complement activation (CR2-Crry) mitigates the effects of LPS-induced pathology and preterm birth. Complement inhibition increased latency to delivery, mean gestational age at delivery, and average number of viable pups. Furthermore, the improved delivery outcomes seen with CR2-Crry treatment correlated with a reduced inflammatory response in maternal tissue and in fetal brain tissue in terms of reduced complement activation, reduced pro-inflammatory cytokines, and reduced macrophage recruitment. These data indicate that complement inhibition represents a potential therapeutic strategy for preventing preterm birth. The localization of complement inhibition by a site-targeting approach reduces the possibility of unwanted off-target effects.

## 1. Introduction

Preterm birth (PTB), defined as delivery before 37 weeks’ gestation, is the leading cause of neonatal morbidity and mortality, and occurs in approximately 12% of deliveries in the United States [1,2]. Common complications associated with PTB include neonatal sepsis, pulmonary hypoplasia, cerebral palsy, necrotizing enterocolitis, intraventricular hemorrhage, and death. Spontaneous preterm labor is found to be highly associated with ascending infection, as studies have demonstrated higher rates of intra-amniotic infection correlate with earlier preterm delivery [3,4]. Further, it is well established from multiple clinical and pre-clinical studies that maternal-fetal inflammatory activation following microbial-induced preterm labor is associated with neonatal pathological outcomes [5,6,7]. While this association between maternal-fetal inflammation, subsequent preterm delivery, and gestational age-driven sequelae is well established, prevention and abating efforts have remained a clinical challenge [8].

Complement, a component of the immune system, plays a central cascading role in orchestrating inflammatory responses and is also involved in homeostatic mechanisms of labor induction and parturition [9]. More specifically, previous human and mouse studies have identified complement as having a role in the initiation of labor and further have found that aberrant activation of complement correlates with PTB outcomes [10,11,12]. Mechanistically, complement has been implicated in initiating PTB via the recruitment of macrophages that ultimately lead to collagen degradation and cervical remodeling [11,12]. More recently, complement component C5a has been found to have a role in myometrial contractions and increased C5a levels are seen in the myometrium of mice that received LPS injections compared to age-matched controls [12]. Further, C5a was found to contribute to fetal cortical brain injury, including disruption of cortical dendritic and axonal cytoarchitecture in a mouse model of PTB [13]. Altogether, there is increasing evidence suggesting that the complement system is implicated in the induction of PTB and that it has a lasting inflammation-related impact on fetal development. While previous work has implicated complement activation in the progression of inflammatory-induced preterm labor, further characterization is needed to provide translational perspective in a clinical setting [9,10].

Here, we provide additional evidence of a role for complement in PTB. We show complement-mediated inflammation affects cervical and uterine tissue and leads to aberrant cellular changes and increased preterm delivery. We further show that following inflammation-induced preterm birth, there is a pro-inflammatory response in terms of modified cytokine and chemokine production in both maternal tissue as well as in the fetal brain. We also show that a complement inhibitor targeted to sites of complement activation (CR2-Crry) mitigates both maternal and fetal pathological outcomes, including a reduction in macrophage recruitment, and results in increased latency to delivery. CR2-Crry interferes with the formation and stability of C3 convertase, a central enzymatic complex assembled following complement activation via any of the three main pathways. By inhibiting C3 activation, CR2-Crry blocks the generation of all major complement activation products with biological function, which are formed downstream of C3 activation [14,15].

Currently, there are few clinical management tools available for prevention of preterm birth, including tocolysis, vaginal progesterone, and cervical cerclage, all which have modest effects on reduction in preterm birth rates [8,16]. Our data indicate that complement inhibition represents a potential novel therapeutic preventative strategy for preterm labor and the associated neonatal complications of preterm birth. To note, a targeted complement inhibitor that targets the same ligand as the murine C3 inhibitor CR2-Crry used in the current study, has been shown to be well tolerated in phase 1 trials, and is now in phase 2 clinical development [17].

## 2. Material and Methods

### 2.1. Study Design

An initial experiment was performed to evaluate the relationship between LPS administration, complement deposition, and cytokine response. All animals underwent laparotomy and received intrauterine LPS administration as described below. Animals were then euthanized at various timepoints and tissues collected for processing. This initial experiment was performed to evaluate the latency between LPS administration, complement deposition, and immune-mediated cytokine response. To evaluate the role of complement inhibition in preterm birth, two experimental groups were created. On day E15 of gestation, animals were either randomized to receive tail vein injection of CR2-Crry at a dose of 20 mg/kg, or an equal volume of PBS, at 1 h and again at 9 h after LPS injection. Dose and timing were based on our previous studies with CR2-Crry in other models [14]. Animals were then evaluated every 6 h for evidence of labor. Pups delivered before E19 were considered preterm, while dams that delivered between E19 and E21 were considered term deliveries. Daily weights were obtained. Mothers were euthanized by isoflurane overdose and decapitation and feti removed and perfused as previously described, with tissues collected for analysis [18]. The study was performed in accordance with ARRIVE guidelines.

### 2.2. Animal Husbandry and Care

Experiments were performed in accordance with the Medical University of South Carolina (MUSC) Institutional Animal Use and Care Committee (IACUC) guidelines (Protocol ID IACUC-2020-01114). C57BL/6 timed-pregnant mice were purchased from Charles River Laboratories (Raleigh, NC, USA). Animals were shipped 10 to 12 days after mating and acclimated in a temperature and light-controlled unit for 2–3 days until the day of surgery. Animals were exposed to 12 h of light/dark cycles, with access to a high-fat diet as recommended by institutional IACUC. The timing of pregnancy was determined by presence of vaginal plug in the Charles River Laboratory, with vaginal plug being day 0 of pregnancy. The duration of a typical murine pregnancy ranges between 19 and 21 days. All experiments conducted in this study utilized C57BL/6 to ensure controlling of strain variation involving maternal and neonatal murine outcomes.

### 2.3. Model of Preterm Birth

A well-studied inflammatory model of preterm birth utilizing lipopolysaccharide (LPS) was employed. This model induces preterm birth in >95% of animals with minimal maternal mortality, and also closely mimics the clinical scenario of a localized intrauterine infection [19]. On day 15 of pregnancy, pregnant mice were placed under a mask with continuous isoflurane administration to obtain deep anesthesia. Toe pinch was used to confirm anesthetic efficacy. A vertical abdominal laparotomy was performed, the left uterine horn was exteriorized, and 25 mcg of LPS (Escherichia coli serotype 0111:B4, Calbiochem, LaJolla, CA, USA) was injected into the myometrium between the two most distal gestational sacs, with care to avoid entry into the amniotic cavity. The peritoneum was then closed with 3-0 vicryl suture, and skin closed with 4-0 vicryl in a running fashion. Following surgery, animals were placed on a heating pad and returned to their cages. They were closely observed for signs of pain and postoperative complications. Animals were weighed daily and evaluated every 6 h until delivery for evidence of preterm birth. Pup viability was determined by presence of movement and responsiveness to handling immediately after birth and 1 h postpartum [20].

### 2.4. Tissue Processing

Animals were euthanized and underwent cardiac perfusion with cold PBS (to flush systemic and unbound complement) followed by 4% paraformaldehyde. Tissues were collected for analysis including uterus, cervix, and pup brains. Tissues were embedded in Tissue Plus Optimal Cutting Temperature Compound (OCT) (23-730-571, Fisher Healthcare, Houston, TX, USA) and frozen at −80 °C. Sections from cervical tissue were cut in 40 um size axial sections using a freeze-mount cryostat. Cut sections were stored in PBS until staining.

### 2.5. Immunofluorescence Staining and Imaging

Immunofluorescence (IF) staining for macrophages (Iba1) and complement (C3) was performed on uterine and uterine-cervical tissue collected at intrapartum. This timing allowed us to assess macrophage recruitment in close temporal proximity to complement activation and preceding or coinciding with preterm labor. Axial sections of the cervix and uterus were stained using standard IF protocols as previously described [21]. All imaging and analyses were performed by lab personnel blinded to experimental samples. For Iba1 and C3 IF staining, high-resolution imaging was performed using a Zeiss LSM 880 confocal microscope (Zeiss, Carl Zeiss Microscopy, LLC, White Plains, NY, USA) at 40× with water-media overlay. Iba1 was specifically used in this study as it is a common and well-validated pan-macrophagic marker that is expressed under normal physiological and pathological conditions. Three randomized images were taken and averaged together for two randomly selected tissue slices per sample. Iba1 and C3 were quantified by calculating the total integrated density (product of Area and the average signal intensity per pixel as a Mean Gray Value) using NIH ImageJ (FIJI). All staining included negative control images (using secondary antibodies only) to correct for underlying auto-fluorescence. Primary antibodies used for staining were anti-C3 (Abcam, Cat. #: ab11863, 1:200) and anti-Iba1 (Abcam, Cambridge, UK, Cat. #: ab5076, 1:200). Secondary antibodies utilized were all donkeys and were anti-rat Alexa Fluor 555 nm (Abcam, Cat. #:ab150154, 1:200) and anti-goat Alexa Fluor 647 nm (Invitrogen, Carlsbad, CA, USA, Cat. #: A32849, 1:200).

### 2.6. Recombinant Protein

CR2-Crry is a recombinant fusion protein composed of the complement receptor 2 targeting domain linked to the complement regulatory protein Crry. The preparation and purification of CR2-Crry has been described previously and briefly consists of protein expression by stably transfected CHO cells, and purification from supernatant by anti-CR2 affinity chromatography [15]. The complement inhibitory activity of the recombinant protein was verified using a zymosan assay, as previously described [22]. The proteins were stored at −80 °C, and once thawed stored under sterile conditions at 4 °C.

### 2.7. Cytokine Assays

Multiplex ELISA was performed on uterine and fetal brain tissue collected at intrapartum. Following euthanasia of animals, tissues were collected and stored at –80 degrees. Tissues collected for cytokine quantification included maternal uterus and pup brains. Per protocol, tissue homogenates were prepared, and cytokine production was determined by Pro-inflammatory Multiplex assay via Eve Technologies, Calgary, Canada. Cytokine analysis included GMCSF, interferon gamma, Interleukin (IL)-1 beta, IL-2, IL-4, IL-6, IL-10, monocyte chemotactic protein (MCP-1), and TNF alpha.

### 2.8. Statistical Analysis

The experimental sample size was determined using power analysis and sample size estimation, performed through G*Power 3.1.9.2 tool as required by the IACUC from MUSC. This tool allowed us to calculate the minimum sample size to detect the difference between group means specified by the user. Based on our data, an effect size of 1.6 is anticipated between an effective C inhibitor treatment and vehicle control and preterm birth. Using an alpha = 0.05 and corrected αc = α/(number of primary comparisons) = 0.05/(2 primary comparisons) = 0.025, we calculated a sample size of 7 mice per group with a computed power of greater than 80% for histological/immune/cellular analysis. Statistical analysis was performed using GraphPad Prism 8.0 (GraphPad Software, San Diego, CA). Details of statistical tests used for different analyses are described in figure legends. Student’s *t*-test was employed for parametric data and Mann–Whitney test for non-parametric data or specified in figure legend otherwise. All data in manuscript are represented as mean with standard error of mean or median with interquartile range where appropriate and *p* values < 0.05 were considered significant.

## 3. Results

### 3.1. Complement Activation Increases over Time in Model of Preterm Birth

**Figure 1:**
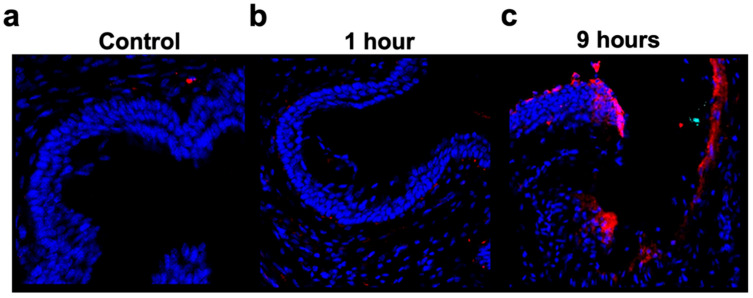
Deposition of complement (C3) in murine cervical tissue after LPS administration. Representative 40× immunofluorescent imaging of C3, captured at different timepoints following LPS administration; no quantitative analysis performed. (**a**) Naïve cervical tissue showing minimal C3 deposition. (**b**) Cervical tissue collected 1 h after LPS administration showing minimal, but increased C3 deposition compared to naive control tissue. (**c**) Cervical tissue collected 9 h after LPS administration showing increased C3 deposition compared to both naive control and 1 h post-LPS administration tissue.

Previous work has implicated a role for complement in the pathology of cervical remodeling and preterm birth [9,11]. Using an inflammatory model of preterm birth utilizing LPS, we investigated C3 deposition within uterine tissue at different timepoints after administration. Following the administration of LPS directly into the maternal uterus on E15, evidence of C3 deposition was seen in the cervical tissue as early as 1 h after LPS administration, with notably higher levels of C3 deposition at 9 h (Figure 1). These data provide the rationale for investigating the use of CR2-Crry in this model, since CR2-Crry targets specifically to sites of complement activation (C3 deposition) which is present at both the 1 and 9 h CR2-Crry administration timepoints used in this study [9,12].

### 3.2. Effect of CR2-Crry on Preterm Birth Delivery Outcomes and Cellular Changes

**Figure 2:**
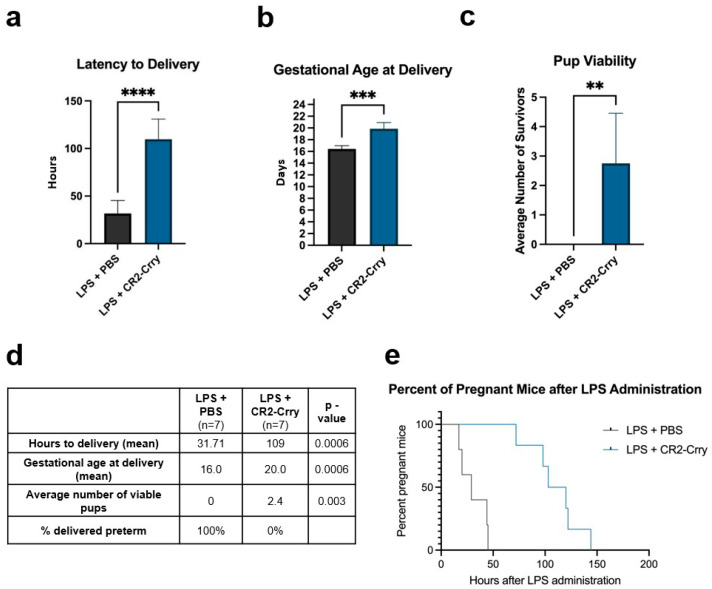
Complement inhibition increases latency to delivery and completion of full-term deliveries. Following LPS administration and subsequent treatment with CR2-Crry or vehicle, multiple parameters of length and completion of successful term deliveries were compared. CR2-Crry (**a**) increased latency to delivery, (**b**) mean gestational age at delivery, and (**c**) average number of viable pups. (**d**) Summary of pregnancy outcomes. n = 7 vehicle and n = 7 CR2-Crry-treated. Hours to delivery comparison made with Welch’s *t*-test. Gestational age at delivery and number of viable pups’ comparison made with Mann–Whitney test. ** *p*< 0.01, *** *p*< 0.001, **** *p*< 0.0001. Error bar = mean ± SD. (**e**) Kaplan–Meier curve showing that 50% of CR2-Crry-treated dams were pregnant at 100 h after LPS administration while 0 vehicle-treated dams remained pregnant.

We next determined the effect of CR2-Crry in our model to investigate the role of complement, and specifically C3 activation, in cervical remodeling in a clinically relevant setting. We injected intrauterine bacterial endotoxin (LPS) to timed pregnant C57BL/6J dams on embryo day 15 (E15), followed by treatment with CR2-Crry or PBS vehicle control. Vehicle-treated dams delivered in approximately 31 h (1.3 days) compared to 109 h (4.3 days) for CR2-Crry-treated dams, *p* = 0.0006, (Figure 2a,d). We also found that CR2-Crry-treated animals were more likely to go to full term, with a median gestational age at delivery of 20.0 days, compared to 16.0 for vehicle-treated dams (*p* = 0.0006) (Figure 2b,d). There was an average of 2.4 viable pups in the CR2-Crry-treated animals compared to 0 viable pups in vehicle-treated animals (*p* = 0.003) as well as treatment with CR2-Crry resulting in all treated animals completing full-term deliveries. (Figure 2c,d). A Kaplan–Meier curve was generated, demonstrating 50% of CR2-Crry-treated dams were still pregnant at 100 h after LPS administration, compared to 0 in the vehicle-treated group (Figure 2e).

**Figure 3:**
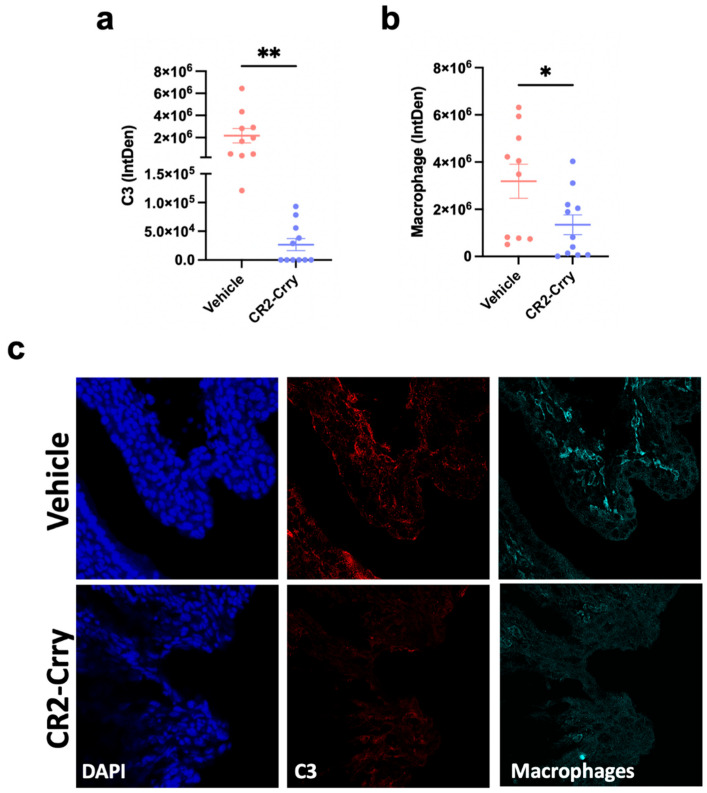
CR2-Crry treatment reduces complement deposition and macrophage recruitment in cervical tissue. Sections stained by immunofluorescence for C3 and macrophages (Iba1). (**a**) Quantification of C3 deposition following LPS administration showing significance increase in vehicle-treated vs. CR2-Crry-treated animals. (**b**) Quantification of macrophage recruitment following LPS administration showing significant increase in vehicle-treated vs. CR2-Crry-treated animals, which correlated with levels of C3 deposition. (**c**) 40× representative images of C3 (red) and Iba1 (teal) in maternal dam cervical tissue. n = 10 vehicle and n = 11 CR2-Crry-treated. Student’s *t*-test. * *p* < 0.05, ** *p* < 0.01. Error bars = mean ± SEM.

In models of PTB, complement activation leads to aberrant macrophage activity involving the release of MMP-9, an enzyme that is known to degrade collagen, leading to cervical dilation and preterm delivery [12,23]. We therefore investigated the effect of CR2-Crry on C3 deposition and macrophage infiltration within the cervix of pregnant dams. CR2-Crry treatment markedly reduced cervical C3 deposition compared to vehicle treatment (*p* = 0.0024) (Figure 3a,c). Additionally, vehicle-treated dams demonstrated significantly higher levels of macrophage infiltration within cervical tissue compared to CR2-Crry-treated animals, *p* = 0.0367 (Figure 3b,c).

### 3.3. Mitigation of Pro-Inflammatory Maternal and Fetal Response in Model of Preterm Birth

**Figure 4:**
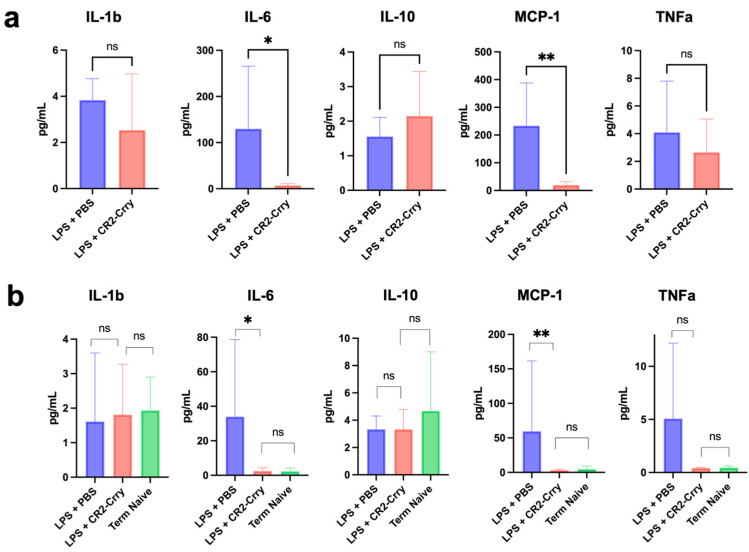
CR2-Crry treatment reduces pro-inflammatory maternal and fetal response. Cytokine levels within homogenized maternal uterine tissue and fetal brain tissue for IL-1b, IL-6, IL-10, MCP-1, TNFa. (**a**) Quantification of pro-inflammatory cytokines in maternal uterine tissue comparing vehicle-treated (n = 7) with CR2-Crry-treated (n = 7) maternal dams following LPS administration. There was a significant reduction in IL-6 and MCP-1 levels with CR2-Crry treatment. All comparisons made with Mann–Whitney test and Error bars = median ± SD, with exception of MCP-1 where comparison was made with Welch’s *t*-test. * *p* < 0.05, ** *p* < 0.01. (**b**) Quantification of pro-inflammatory cytokines in fetal brain tissue comparing. Fetal brains were collected from naïve (n = 8), vehicle-treated (n = 13), or CR2-Crry-treated (n = 9) dams following administration of maternal LPS administration. A significant reduction was found in IL-6 and MCP-1 levels in fetal pups from maternal dams treated with CR2-Crry with levels similar to that seen in full-term naïve pups. All comparisons made with Mann–Whitney test and Error bars = median ± SD, with exception of IL-1b (CR2-Crry and Naive) where comparison was made with Welch’s *t*-test. * *p* < 0.05, ** *p* < 0.01. Error bars = mean ± SD.

In term and preterm deliveries, intrauterine inflammation has been associated with an increased risk of neonatal neurological pathology [1,13,24]. Previous work has also demonstrated that Maternal Immune Activation measured as intrauterine inflammation in an LPS model can give rise to a fetal brain inflammatory response that results in long-term neurodevelopmental abnormalities [25]. In order to investigate the effects of CR2-Crry treatment on maternal and fetal inflammation, cytokine levels (IL-1B, IL-6, IL-10, MCP-1, and TNFa) were analyzed within both intrapartum uterine and fetal brain tissue. Compared to vehicle, intrapartum uterine tissue of CR2-Crry-treated dams demonstrated a reduction in IL-6 (109.6 pg/mL vs. 7.10 pg/mL, *p* value 0.0221) and MCP-1 (145.5 pg/mL vs. 18.49 pg/mL, *p* value = 0.0012) (Figure 4a). There was no difference in IL-1 beta (3.45 pg/mL vs. 1.64 pg/mL, *p* = 0.29), IL-10 (1.6 pg/mL vs. 2.1 pg/mL, *p* = 0.75), and TNF alpha (3.34 pg/mL vs. 1.62 pg/mL, *p* = 0.46) between treatment groups. Similarly, within fetal brain tissue, IL-6 and MCP-1 were significantly higher in the offspring of dams that did not receive complement inhibition (33.98 pg/mL vs. 2.47 pg/mL, *p* = 0.04 and 145.5 pg/mL vs. 18.5 pg/mL *p* = 0.0012), respectively. When comparing fetal neural inflammation in dams treated with CR2-Crry to gestational-age matched naïve pups, IL-6 levels (2.48 pg/mL vs. 2.29 pg/mL, *p* = 0.85) and MCP-1 levels (3.19 pg/mL vs. 3.53 pg/mL, *p* = 0.65) were similar between groups (Figure 4b).

## 4. Discussion

Preterm delivery is the leading cause of neonatal morbidity and mortality in the United States and is responsible for significant sequelae including neonatal sepsis, cerebral palsy, intraventricular hemorrhage, and postnatal demise [26,27]. The common pathway of preterm delivery coalesces in myometrial contractions, cervical remodeling, and premature rupture of membranes, and is found to be highly impacted by inflammatory responses. Intrauterine inflammation is strongly associated with PTB, with histologic evidence of inflammation in more than 50% of placentas with extreme prematurity [28]. While inflammation is strongly implicated in PTB, inflammatory mechanisms leading to preterm delivery are not well characterized, thus leading to a lack of therapeutic options [8]. In this pathological setting, the complement system, a component of the immune system, has been implicated in preterm delivery in both animal and human studies [3,29,30].

Complement can play a pivotal role in preterm birth given its association with smooth muscle contractions, cervical collagen remodeling, and immune cell recruitment [12,23]. In the current study, we found complement deposition in cervical tissues as early as 1 h after LPS administration with increased deposition at 9 h, supporting a current hypothesis that ascending infection from the vagina recruits complement leading to its activation in the cervical stroma [9]. Herein, we employed a complement inhibitor, CR2-Crry, to mitigate complement deposition. CR2-Crry specifically targets C3d deposition at sites of complement activation. The C3d opsonin is a C3 cleavage product of the complement cascade, and remains present on tissues for an extended period of time, thus rationalizing its use as a target for localizing complement inhibition [15]. Additionally, the use of site-targeted complement inhibition is able to mitigate unwanted and potentially dangerous off-target effects by obviating the need to systemically inhibit complement. This is an important consideration since complement has important roles in host defense as well as various homeostatic and reparative functions [14,15]. Of note, it has been demonstrated in mouse models that complement activation is not required for normal term delivery, but is upregulated in preterm birth, making it an optimal target under pathological conditions [11].

Complement has an important role in the recruitment and activation of macrophages and neutrophils [11,31]. In the setting of preterm delivery, macrophages have been found to stimulate the release of metalloproteinases (MMP-9) that degrade collagen and ultimately lead to cervical distension and dilation [11,12,32]. Studies in humans have also shown increased macrophage recruitment in both term and preterm deliveries [33]. When evaluating maternal tissues, we found complement deposition correlated with increased macrophage recruitment, and that dams treated with CR2-Crry showed a significant reduction in both complement deposition and macrophage recruitment in cervical tissue, which is associated with cervical ripening and distension as described above [12]. Moreover, animals treated with CR2-Crry had significantly improved pregnancy outcomes, including greater latency to delivery, greater gestational age at delivery and an increase in the number of viable pups. We show recruitment of macrophages in a complement-dependent manner, but did not investigate macrophage subpopulations (residential, infiltrating and activation status) or the temporal landscape, which we acknowledge is a limitation to the current study. We also did not address the source of C3 in terms of peripheral vs. cellular and cell type. These are important considerations and worthy of further study, but are outside the current scope. Of note, in the uterine-cervical environment, C3 is primarily produced by decidual stromal cells, trophoblasts, uterine epithelial cells, as well as residential macrophages potentially also contributing to C3 production, particularly in response to inflammatory stimuli [12,34].

We also found that dams treated with CR2-Crry had a significant reduction in the pro-inflammatory cytokines IL-6 and MCP-1. IL-6 is a key mediator of acute inflammatory responses and tissue injury, and MCP-1 functions to recruit monocytes and macrophages to sites of inflammation [3]. Multiple studies have demonstrated increased concentrations of pro-inflammatory cytokines in the amniotic fluid and umbilical cord blood of patients with preterm delivery [7,35,36]. The degree of histologically confirmed intrauterine infection positively correlates with increasing amniotic fluid concentrations of IL-6 [35,37]. More severe histologic features include funisitis and umbilical arteritis, which demonstrates that the inflammatory response has progressed from the maternal compartment to the fetal compartment, and has significant postnatal sequelae [3,37]. Our study also demonstrated that pups of vehicle-treated dams showed a significant increase in IL-6 concentrations within the fetal brain, and this fetal brain inflammatory response was mitigated to levels similar to naïve fetal brain concentrations by CR2-Crry treatment. This “fetal inflammatory response” (FIRS) has been clinically defined as elevated plasma concentrations of IL-6, thus the reduction in IL-6 with complement inhibition identified in our study has meaningful implications [3]. Elevated concentrations of IL-6 have been linked to adverse postnatal outcomes including pulmonary injury, renal dysfunction, gut inflammation, and neural injury [3,7,29]. Most recently, IL-17a, a downstream product of IL-6, from a subset of T helper cells was found to cause cortical defects and is associated with autism-spectrum disorder behavior in offsprings, providing relevance to the finding that the mother’s immune system during pregnancy impacts fetal outcome [25]. In support of our findings on the complement-dependent impact on preterm birth and fetal inflammation, previous studies have demonstrated that Complement C5a in the CSF of human neonates is associated with preterm birth [38]. Preclinical models that modulate C5a were also found to be protective against fetal cortical brain injury in PTB mice, providing further evidence of the potential benefits of CR2-Crry in the setting of PTB [13]. Additionally, preterm infants are vulnerable to white matter injury and germinal matrix hemorrhage (intraventricular hemorrhage) due to immaturity of the vascular system and cortical tissue [29,39]. Further supporting the impact of maternal inflammation on FIRS, recent studies have found that complement-mediated inflammation plays a critical role in the pathological sequalae of germinal matrix hemorrhage, including the development of post-hemorrhagic hydrocephalus and periventricular leukomalacia [18,40,41].

The current study shows that site-targeted complement inhibition has significant therapeutic potential by delaying preterm delivery and mitigating post-natal sequelae associated with preterm birth and additionally protects against pathological sequelae associated with LPS-mediated fetal neural inflammation. While our findings offer initial evidence supporting a complement-mediated injury mechanism that can be treated by pharmacological intervention, there are nonetheless limitations to our study. Lacking is an in-depth examination of immune mechanisms; a more comprehensive profiling of the inflammatory microenvironment at the maternal, fetal, and neonatal level would broaden the relevance of the current findings. Future studies will build upon and better delineate the local inflammatory milieu, as well as better define the complement-dependent immune landscape within maternal tissue and the effect of complement modulation on PTB and neonatal brain injury. This could be addressed by more detailed immunophenotyping and spatial mapping of the complement activity and associated inflammatory mediators. We did not observe significant effects of CR2-Crry treatment alone in unmanipulated mice, consistent with the previous literature, thus we do not expect any adverse off-target effects of CR2-Crry. Future work will be able to determine safety and efficacy together with timing and dosing of complement inhibition. Of note, a human-targeted complement C3 inhibitor that targets the same ligand as the murine CR2-Crry used here, has been shown to be well tolerated in phase 1 trials, and ready for phase 2 clinical development [17].

## 5. Conclusions

Our study demonstrates that CR2-Crry not only reduces cervical ripening and preterm delivery but also potentially reduces the fetal inflammatory response. In our model, LPS administration initially stimulated a maternal increase in cytokine production, followed by a delayed fetal production of cytokines, suggesting an opportunity for intervention. This window may represent a time span where CR2-crry can be administered to reduce preterm birth and fetal inflammatory sequelae. In conclusion, using a well-studied murine model of preterm delivery that closely resembles the clinical scenario of intrauterine inflammation, we found that CR2-Crry prevents macrophage recruitment in the cervix, decreases production of pro-inflammatory cytokines in both maternal and fetal tissues, and ultimately decreases preterm birth rates.

## Data Availability

All data generated and/or analyzed during the current study are included in this published article. Materials described in this article that were made in the laboratory will be made available upon request under a materials transfer agreement.
